# Phytotoxic Effects of Polystyrene Microplastics on Growth Morphology, Photosynthesis, Gaseous Exchange and Oxidative Stress of Wheat Vary with Concentration and Shape

**DOI:** 10.3390/toxics13010057

**Published:** 2025-01-15

**Authors:** Komal Riaz, Tahira Yasmeen, Kotb A. Attia, Itoh Kimiko, Muhammad Saleem Arif

**Affiliations:** 1 Department of Environmental Sciences, Government College University Faisalabad, Faisalabad 38000, Pakistan; komalriaz1215@gmail.com (K.R.); rida_akash@hotmail.com (T.Y.); 2 Department of Biochemistry, College of Science, King Saud University, P.O. Box 2455, Riyadh 11451, Saudi Arabia; kattia1.c@ksu.edu.sa; 3 Institute of Science and Technology, Niigata University, Ikarashi-2, Nishiku, Niigata 950-2181, Japan; kim@agr.niigata-u.ac.jp

**Keywords:** agricultural pollution, abiotic stress, emerging pollutants, cereal crops, food safety

## Abstract

Microplastics pose a serious ecological threat to agricultural soils, as they are very persistent in nature. Microplastics can enter the soil system in different ways and present different shapes and concentrations. However, little is known about how plants react to microplastics with different concentrations and shapes. To this end, we conducted a factorial pot experiment with wheat (*Triticum aestivum* L.) in which we mixed polystyrene (PS) in different shapes (bead, fiber and powder) with soil at concentrations of 0, 1, 3 and 5%. Although all shapes of PS significantly reduced morphological growth traits, PS in powder shape was the microplastic that reduced plant height (by 58–60%), fresh biomass (by 54–55%) and dry biomass (by 61–62%) the most, especially at the 3% and 5% concentrations compared with 0% PS. Similar negative effects were also observed for root length and fresh root weight at the 3% and 5% concentrations, regardless of shape. A concentration-dependent reduction in the leaf area index (LAI) was also observed. Interestingly, increasing the PS concentration tended to up-regulate the activity of antioxidant enzymes for all shapes, indicating potential complexity and a highly time-dependent response related to various reactive oxygen species (ROS). Importantly, PS at the 5% concentration caused a significant reduction in chlorophyll pigmentation and photosynthetic rate. For the transpiration rate, stomatal conductance and intercellular CO_2_ concentration, the negative effects of PS on wheat plants increased with the increase in microplastic concentration for all shapes of PS. Overall, we concluded that PS microplastics at higher concentrations are potentially more devastating to the physiological growth and biochemical attributes of wheat, as evidenced by the negative effects on photosynthetic pigments and gas exchange parameters for all shapes. We recommend further research experiments not only on translocation but also on tissue-specific retention of different sizes in crops to fully understand their impact on food safety.

## 1. Introduction

Understanding the response of soil ecosystems to any type of disturbance is of great importance in predicting the ecological, economic and social consequences of environmental disturbances. Microplastics, <5 mm by-products of meso- and macro-plastics, have been detected ubiquitously in most terrestrial biomes [1,2]. Recent reports of microplastic littering even in one of the world’s most remote areas have raised serious concerns about health and the functioning of the ecosystem [3,4]. Despite growing awareness of plastic pollution, global plastic production has alarmingly surged to 400.3 million metric tons, driven by rapid urbanization and economic activity [5]. Once plastic enters the environment, it has devastating consequences for the sustainability of ecosystems and human health due to the incessant formation of microplastic particles [6].

The global agricultural system is now facing a stern sustainability challenge, as arable soils have increasingly become a hotbed of microplastic pollution via numerous pathways and inputs [7,8]. The accumulation of microplastics in agricultural soils is a rather complex problem due to the non-point source entry of the plastic particles from applied fertilizers, soil amendments, mulching, tire abrasion, irrigation and flooding [9,10]. The majority of microplastics in agricultural soils are of secondary origin, arising from the breakdown of larger plastic waste products [11]. Although microplastics can occur in many shapes and with different physical and chemical properties, research on shape-specific microplastics in agricultural soils has mostly focused on fibers, fragments and films [12]. There is currently little information on ecotoxicological investigations and studies on the accumulation of microplastic beads and powders in agricultural soils.

With the increasing threat of microplastics in agroecosystems, crops are already or will soon be subject to harmful exposure, resulting in reduced plant growth and development [13]. Nevertheless, the negative effects of microplastics on plants are predominantly either type- or dose-dependent [14,15]. Therefore, resistance to the suppressive effects of microplastics is very important for plants to either survive and/or adapt under stressful conditions. Plants possess resistance traits, including improved morphological growth, an active antioxidant enzyme system, efficient photosynthetic pigmentation, and optimized gas exchange, which are crucial adaptations enabling them to withstand widespread microplastic pollution [16,17]. However, plant roots are the first causalities due to their direct interaction with microplastic particles, as the latter can be adsorbed on root hairs and thus impair root growth. In leaves, on the other hand, microplastic contamination most likely causes oxidative stress, which reduces leaf growth and photosynthesis. Intriguingly, the phytotoxic effects of microplastics with varying types, concentrations and shapes on the growth and development of plants remain unclear.

Polystyrene (PS) accounts for over 7% of the global plastics economy, with an estimated annual production of 19.68 million metric tons in 2023 [18]. The widespread production and use of PS in packaging products, food containers, and toys has inevitably led to the release of tiny plastic particles into the environment [19]. In addition, PS microplastics have been shown to have high affinity to persist in the environment for long periods of time, possibly hundreds of years, and thus pose a major ecotoxic threat to ecosystems [20]. Most importantly, PS microplastics are easily transported by water currents due to their low density, thus making them some of the most widespread contaminants accumulating in aquatic ecosystems worldwide [21].

Studies have shown extensive effects of PS on plant growth and development, with the properties of PS microplastics having the greatest impact [22]. For example, certain PS microplastics can stimulate plant growth by increasing root length and photosynthesis and alleviating oxidative stress [23]. In contrast, higher PS concentration can lead to a dampened growth response in plants, resulting in the formation of ROS and reduced water and nutrient uptake [24]. Obviously, in addition to microplastics and the plant species used, the prevailing climatic conditions, soil properties, fertilization and irrigation can also influence various plant growth characteristics.

Wheat (*Triticum aestivum* L.) is one of the most important cultivated plants in the world in terms of its antiquity and its importance as food for humankind. While it has overcome numerous growth and productivity challenges in the past, new abiotic stress factors have emerged in the last decade, particularly microplastic pollution. The individual and combined effects of microplastics, in conjunction with other abiotic stressors, could be an ominous threat for achieving future food security goals, as crop yields are increasingly constrained worldwide. It is, therefore, imperative to study the response of the wheat plant to these stressors in order to better understand the impending problem. The response of wheat growth to different types and concentrations of microplastic pollution has already been studied. However, the response of wheat growth to different addition rates and shapes of microplastics, especially PS, still needs to be investigated.

Therefore, we conducted a pot study using wheat as a test crop to further investigate the potential effects of PS microplastics. Our main objectives were as follows:

1. Investigate the concentration-dependent effects of PS microplastics on the morphological, physiological and biochemical growth characteristics of wheat.
2. Determine the phytotoxic relevance of different shapes of PS microplastics for wheat at different concentrations.

## 2. Materials and Methods

### 2.1. Microplastic Materials

In this study, we used polystyrene microplastic (PS), representing three microplastic shapes: bead, fiber and powder. PS bead and powder were supplied by Pak-Petrochemical Industries, Lahore, Pakistan. We prepared microfibers from PS beads by flattening the beads with a hammer and then treating them with liquid nitrogen for 10 min to make PS more brittle. A ball mill containing the frozen PS particles was operated at 45 rpm for 5 min, and the process was repeated until the desired size (15–25 µm) of PS microfibers was achieved. We used PS powder with a size of 5–7 µm and PS beads with a size of 5–10 µm. The mean particle size of each shape was determined by using a Malvern Mastersizer 2000 laser diffraction system (Malvern Instruments Ltd., Worcestershire, UK). The surface potential of all microplastic materials, expressed as the zeta potential, was measured by using a Zeta-Plus analyzer (Zetasizer Nano ZS90, Malvern Instruments, Worcestershire, UK). The surface functional groups associated with PS of various shapes were characterized by Fourier transform infrared spectrometry (FTIR; 670-IR + 610-IR; Varian, Salt Lake City, UT, USA). All microplastic material was washed with methanol to remove impurities and then dried at room temperature for 24–48 h. The physical properties of polystyrene microplastics of different shapes used in this study are listed in Table 1.

### 2.2. Soil Sampling and Preparation

We collected sandy loam soil (Haplic Calcisols; 58.12% sand, 19.73% clay, 22.25% silt) from an irrigated arable field (wheat–maize–maize) in Diyalgarh, Faisalabad, Pakistan (73°09′ E, 31°34′ N). The topsoil (0–20 cm) was selected for sampling because of its importance for plant nutrients and biological activity. We collected soil samples in bulk to ensure that a sufficient amount of soil is available for the experiment. Composite soil samples were spread on a clean polyethylene sheet for air drying. The air-dried soil was sieved through a 2 mm mesh to remove stones and debris.

### 2.3. Experimental Design

A pot trial was conducted in the botanical garden of Government College University Faisalabad, Pakistan. For the potting experiment, soil samples were prepared with varying concentrations of microplastic contamination at 0%, 1%, 3%, and 5% (*w*/*w*) of PS in three distinct shapes (bead, fiber and powder). Each pot was filled with 8 kg of soil pot^−1^, and treatments were applied in a completely randomized design in a two-factorial arrangement (PS addition rate and PS shape) with four replicates. Before sowing, the pots were allowed to stabilize with the incorporated microplastic for one week. The soil–microplastic mixture in the pots was watered thrice to ensure optimal moisture availability for seed germination. In this study, an indigenous high-yielding wheat variety, Anaj-2017, was used to test the concentration- and shape-dependent phytotoxic effects of PS microplastics. Uniform seeds of good quality were acquired from the seed bank of the Ayub Agriculture Institute (AARI), Faisalabad. Seeds were surface-sterilized with 4% sodium hypochlorite for 5 min and 75% ethanol for 2 min and then rinsed thoroughly with sterile water. Six surface-sterilized seeds were sown in plastic pots. Four uniform plants were selected for the experiment, while the remaining plants were culled after one week of germination. The pots were placed in the sunlight, and their location was changed weekly. The nutrient requirements of the plants were met by fertilizing them with the recommended dose of chemical fertilizers: 120-80-60 NPK kg ha^−1^ in the form of urea, single super phosphate and potassium sulfate. Half of the N and the total dose of P and K were mixed into the soil at sowing, and the remaining N was applied in equal proportions at the time of the first two irrigation operations. After six weeks of growth, two whole plants were harvested from each pot, while the growth of the remaining two plants continued until physiological maturity.

### 2.4. Growth Morphology Parameters

Forty-two-day-old wheat plants were cut at the base of the stem to record growth morphology parameters. At harvest, the above-ground plant and below-ground root samples were separated and washed before plant height and root length were measured by a measuring tape. The fresh weight of the shoots and roots was determined on the same day by using an electric scale. The dry biomass of the above-ground plants was recorded after oven drying at 70 °C for 24 h. From each individual treatment pot, fully expanded leaves were collected and measured for the leaf area index by using a digital leaf area meter.

### 2.5. Oxidative Stress Indicators

For antioxidant enzyme activity, approximately 0.5 g of fresh leaves were crushed and homogenized in 50 mM phosphate buffer (pH 7.8). The ground mixture was then centrifuged at 15,000× *g* for 30 min at 4 °C, and the supernatant was collected for enzyme assays. For superoxide dismutase activity (SOD), the enzyme extract was assayed colorimetrically at 560 nm for the photochemical reduction of nitro blue tetrazolium (NBT) to avoid the formation of the formazan chromophore [25]. For peroxidase activity (POD), a reaction mixture [100 µL enzyme extract + 100 µL H_2_O_2_ + 250 µL 2% guaiacol + 780 µL phosphate buffer] was used to monitor the polymerization of guaiacol to tetraguaiacol colorimetrically by absorbance intensity at 470 nm [26]. For catalase activity (CAT), an enzyme reaction mixture containing enzyme extract, phosphate buffer, H_2_O_2_ and distilled water was subjected to the colorimetric monitoring of the decrease in H_2_O_2_ absorbance at 240 nm [27]. Malondialdehyde content (MDA) was determined as a measure of lipid peroxidation by homogenizing fresh green leaves (0.3 g) in 4 mL of a thiobarbituric acid reaction mixture and then heating them at 95 °C for 30 min. After rapid cooling in an ice bath, the homogenate was centrifuged at 10,000× *g* for 15 min, and the absorbance was measured at 450, 532 and 600 nm to calculate MDA content [28].

### 2.6. Photosynthetic Pigments

Leaf chlorophyll pigments were extracted in a centrifuge tube containing 4 mL of 80% acetone after adding a few drops of liquid N to accelerate the dissolution of leaf chlorophyll. The fully dissolved leaf chlorophyll was recovered after an overnight dark incubation at 4 °C. The absorbance values for chlorophyll-a and -b and total chlorophyll from the supernatant were measured colorimetrically at 663, 645 and 470 nm, respectively [29].

### 2.7. Leaf Gas Exchange Parameters

At the physiological maturity stage, the fully exposed top-third leaf of each treatment pot was used for gas exchange measurement. We monitored the net photosynthetic rate, transpiration rate, stomatal conductance and intercellular CO_2_ concentration by using a LI-COR portable photosynthesis system (LI-6400; LI-COR Biosciences, Lincoln, NE, USA). All the measurements were performed on a clear, bright sunny day between 9:00 and 11:00 am, according to Long et al. [30].

### 2.8. Statistical Analysis

All the statistical analyses were performed with R statistical software in R studio (version 4.2.2, Boston, MA, USA). The data distribution of all response variables was examined for normality and homogeneity by using the Shapiro–Wilk test prior to the analysis. Two-way analysis of variance (ANOVA) with interaction was employed to illustrate the main effects of PS microplastics applied in different concentrations and shapes on wheat morphological growth, antioxidant enzyme activity, photosynthetic pigments and gaseous exchange attributes. Tukey’s HSD multiple mean comparison was used at *p* < 5%. One-way analysis of variance (ANOVA) was used to test the morphological and physiological responses of wheat to PS microplastic application in relation to each shape. The data presented in both the tables and figures include the means of four replicates from the non-transformed data pool.

## 3. Results

### 3.1. Microplastic Characterization

**Figure 1:**
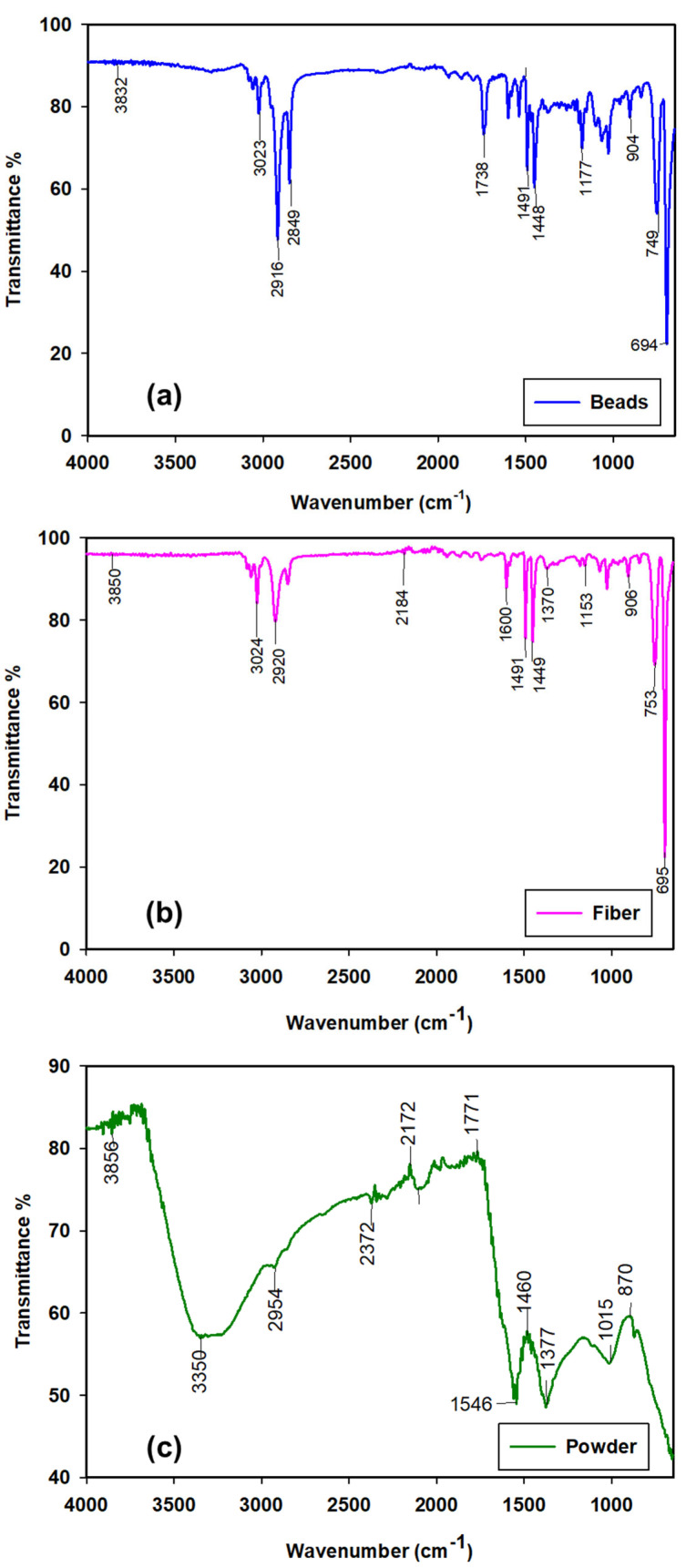
Fourier transform infrared spectrograms of (**a**) bead (**b**) fiber and (**c**) powder microplastics (polystyrene).

In this study, rather than using PS microplastics with defined shape and size, we prepared PS microplastics of heterogenous sizes and shapes to mimic microplastics in the environment. The average size distributions of PS microplastics in the form of powder, bead and fiber were 5–7, 5–10 and 15–25 µm, respectively (Table 1). Among all PS shapes, the powder shape had the lowest density, 0.95 g cm^−3^, whereas the density of bead and fiber microplastics ranged from 1.05 to 1.17 g cm^−3^. The zeta potential of PS with varying shapes ranged from −35 to −40 mV, indicating uniform structural stability of the suspended matrix. The comparison of infrared absorption spectra of PS revealed differences in the functional group across all shapes (Figure 1a–c). The absorption peaks observed at 3023 cm^−1^, 3024 cm^−1^ and 3350 cm^−1^ are attributable to the C-H bending vibrations of PS beads, fiber and powder, respectively. Furthermore, the pronounced C═C stretching in the aromatic structure of beads, fibers and powder is apparent from the peaks at 1448–1491 cm^−1^, 1449–1491 cm^−1^ and 1460 cm^−1^, indicating the presence of a benzene ring structure in all polystyrene materials.

### 3.2. Morphological and Root Growth Response to PS Microplastics

**Figure 2:**
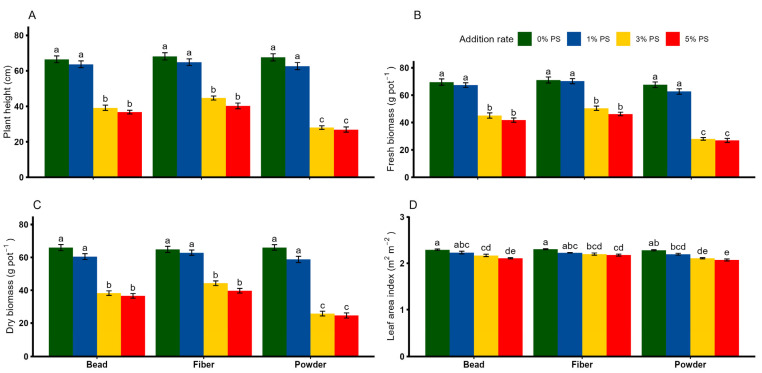
Effects of different shapes and dosages of microplastic (polystyrene) on (**A**) plant height, (**B**) fresh biomass, (**C**) dry biomass and (**D**) leaf area index from wheat experiment. Each bar represents values of four replicates and contains standard errors of means. Bar sharing different letters differ significantly from each other at *p* < 0.05.

**Figure 3:**
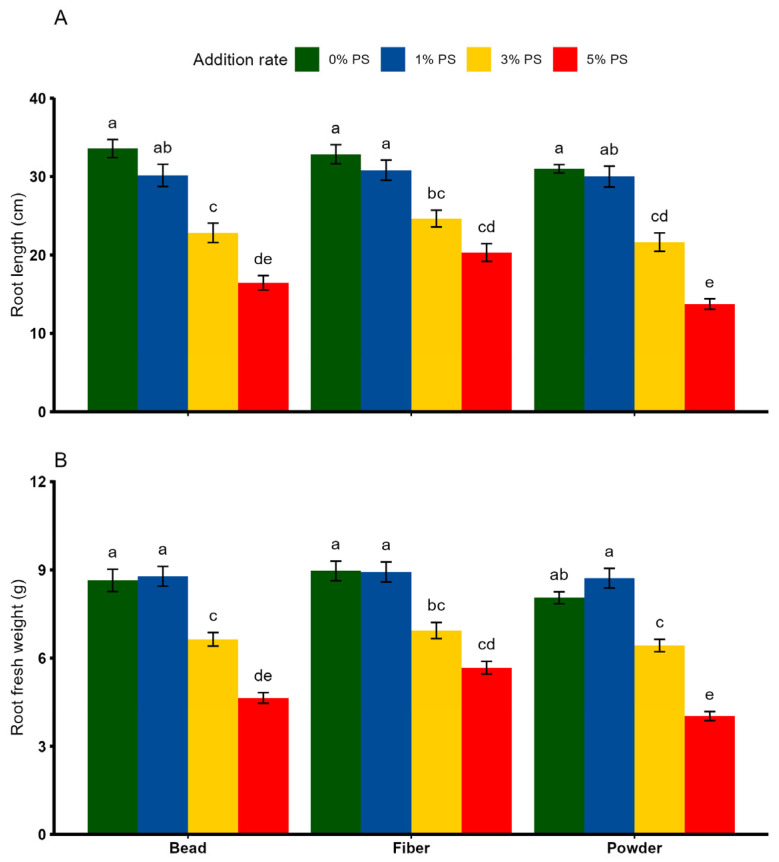
Effects of different shapes and dosages of microplastic (polystyrene) on (**A**) root length and (**B**) root fresh weight from wheat experiment. Each bar represents values of four replicates and contains standard errors of means. Bar sharing different letters differ significantly from each other at *p* < 0.05.

Plant height, fresh biomass, dry biomass and the LAI were significantly affected by PS shape and rate, while their interaction effect was significant only for plant height and biomass parameters (Table 2). Overall, no significant differences in plant height, and fresh and dry biomass were observed between 0% and 1% PS concentrations, regardless of PS shape (Figure 2A–C). However, PS in powder shape exhibited the greatest reduction in plant height (by 58–60%), fresh biomass (by 54–55%) and dry biomass (by 61–62%), particularly at the 3% and 5% concentrations compared with 0% PS (*p* < 0.05). Similarly, PS microplastics of all shapes tended to reduce the LAI at 3% and 5% concentrations compared with the respective control treatments (Figure 2D). With the exception of root fresh weight, all root parameters were significantly affected by shape, rate and shape × rate (Table 2). As far as PS shape is concerned, none of the selected root parameters showed any noticeable change between the 0% and 1% PS concentrations (Figure 3A,B). However, the effect of PS shape on the root growth parameters was only relevant when applied in higher concentrations. Specifically, root growth and root fresh weight were reduced when PS bead, fiber and powder were applied at higher concentrations, between 3% and 5% (Figure 3A,B).

### 3.3. Plant Oxidative Stress Response to PS Microplastics

**Figure 4:**
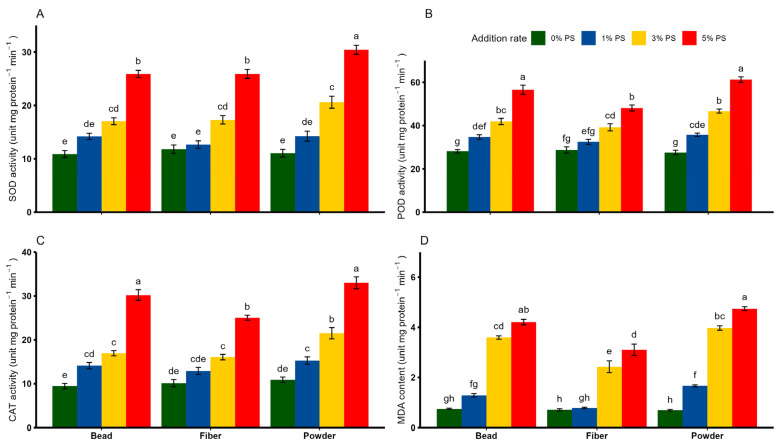
Effects of different shapes and dosages of microplastic (polystyrene) on (**A**) SOD activity, (**B**) POD activity, (**C**) CAT activity and (**D**) MDA content from wheat experiment. Each bar represents values of four replicates and contains standard errors of means. Bar sharing different letters differ significantly from each other at *p* < 0.05.

Overall, we observed a significant individual and interactive effect of PS shape and addition rate on the activities of POD, CAT and MDA (Table 2). Wheat plants exposed to 0%, 1% and 3% PS concentrations showed a down-regulating effect on antioxidant enzymes (SOD, POD and CAT), regardless of PS shape. Contrary to these, a marked increase in antioxidant enzyme activities was observed across all PS shapes, particularly at the 5% PS concentration (Figure 4A–C). The MDA content of wheat plants was statistically identical between 0% and 1% PS bead and fiber. However, 1% PS powder was found to increase MDA content by two times compared with 0% PS (Figure 4D; *p* < 0.05). Among all shapes and rates, the 5% PS concentration elicited a strong stimulatory effect, resulting in the highest MDA content (*p* < 0.05).

### 3.4. Effects of PS Microplastics on Chlorophyll Pigmentation and Photosynthetic Rate

**Figure 5:**
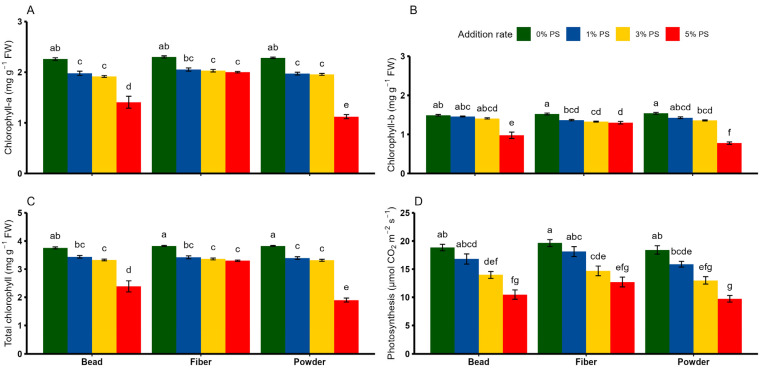
Effects of different shapes and dosages of microplastics (polystyrene) on (**A**) chlorophyll-a, (**B**) chlorophyll-b, (**C**) total chlorophyll and (**D**) photosynthetic rate from wheat experiment. Each bar represents values of four replicates and contains standard errors of means. Bar sharing different letters differ significantly from each other at *p* < 0.05.

We found a significant effect of PS shape, rate and interaction on all chlorophyll parameters, while the net photosynthetic rate revealed a significant individual effect alone (Table 2). Overall, increasing the PS concentration led to reduced chlorophyll-a content compared with the respective control treatment (Figure 5A). In particular, the largest decrease in chlorophyll-a content was observed at the 5% concentration of fiber (−51%), followed by bead (−37%) and powder (−13%), compared with the control (*p* < 0.05). On the other hand, chlorophyll-b content in PS bead did not vary across different concentrations, except for 5% PS, which showed significantly lower chlorophyll content than the control treatment (Figure 5B). A significant decrease in chlorophyll-b content in PS powder (of −49%) and PS fiber (of −14%) was also observed at the 5% concentration compared with the respective controls (*p* < 0.05). As PS bead addition rate increased, a significant suppression of total chlorophyll content was observed, particularly at the concentrations of 3% and 5% (Figure 5C). Compared with the respective control treatments, a significantly lower but identical trend of reduced total chlorophyll content was observed in PS fiber at the 1%, 3% and 5% concentrations. Of all the treatments, PS powder at 5% concentration had the most devastating effect on total chlorophyll content, contributing to a 50% decrease compared with the control treatment (*p* < 0.05). No discernible differences in the net photosynthetic rate were observed between the 0% and 1% PS concentrations in any of the PS shapes (Figure 5D). On the other hand, the negative effects of PS shape were relevant at higher concentrations, with the 5% PS concentration having the strongest effect on the net photosynthetic rate, followed by the 3% concentration, compared with the respective control treatments (*p* < 0.05).

### 3.5. Response of Leaf Gaseous Exchange to PS Microplastics

**Figure 6:**
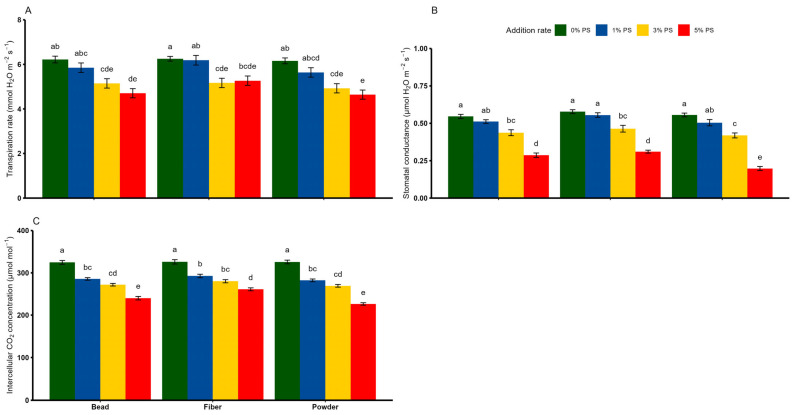
Effects of different shapes and dosages of microplastic (polystyrene) on (**A**) transpiration rate, (**B**) stomatal conductance and (**C**) intercellular CO_2_ concentration from wheat experiment. Each bar represents values of four replicates and contains standard error of means. Bar sharing different letters differ significantly from each other at *p* < 0.05.

We observed an individual effect of PS shape and rate on the leaf transpiration rate and stomatal conductance, whereas their interaction effect was significant only for intercellular CO_2_ concentration (Table 2). In most cases, the transpiration rate, stomatal conductance and intercellular CO_2_ concentration decreased significantly with the increase in PS concentration, regardless of PS shape (Figure 6A–C). However, the impact of PS shape on the leaf gas exchange parameters was relevant at higher PS concentrations, particularly 5% PS. For instance, PS fiber and PS bead led to reductions in the transpiration rate (of −15–−24%), stomatal conductance (of −46–−47%) and intercellular CO_2_ concentration (of −26–−19%), compared with the control treatments (*p* < 0.05). Notably, PS powder at the 5% concentration was the microplastic that decreased leaf gaseous exchange parameters the most, i.e., the transpiration rate (by −25%), stomatal conductance (by −64%) and intercellular CO_2_ concentration (by −30%), relative to the control treatment.

## 4. Discussion

Our findings from the wheat experiment, in which we exposed plants to PS microplastics with different concentrations and shapes, provide evidence that plant morphological growth, antioxidant responsiveness, photosynthesis pigmentation and leaf gas exchange attributes are largely controlled by concentration, while the effects of shape on the plants were relevant at higher PS concentrations. Plant biomass, root length and shoot length are prominent morphological indicators for assessing the overall healthy growth of plants. Wheat plants are known for their conservative early growth, so early shoot growth enhancement is likely to have a substantial pleiotropic effect on root growth traits, contributing to improved competitiveness against any stress factors [31]. The striking differences in shoot and root growth characteristics observed in this study between lower and higher PS concentrations suggest a potentially diverse and complex impact of microplastics, which may be attributed to variations in their size, shape, polymer type and concentration [32,33]. Our plants exhibited higher growth sensitivity and a diminished LAI at the 5% PS concentration, which aligns with findings of numerous prior studies, indicating that higher concentrations of microplastic of smaller size and larger surface area may pose greater toxicity risks [34,35,36]. Also, higher concentrations of these particles typically enable easier penetration and adhesion to root surfaces, thereby limiting water uptake and down-grading plant metabolism, which in turn severely affects root–shoot growth and overall plant development [36,37].

Antioxidant enzymes have been identified as some of the robust defense responses triggered by plants when confronted with various types of stress, such as drought, salinity and microplastics [38]. These enzymes usually employ reactive oxygen species (ROS), which act as key players in a complex cellular signaling system to activate plant defenses and mitigate the damage caused by stress [39]. Our results show that SOD, CAT and POD maintained relatively lower enzyme activity at low PS concentration. Numerous studies have shown that maintaining a low level of ROS can be used as a signal transduction molecule to prevent oxidative damage to a certain extent and regulate plant growth and development [40,41]. In this study, we found that increased PS exposure, especially at the 5% concentration, leads to excessive ROS production, potentially surpassing the scavenging capacity of antioxidant enzymes and MDA levels, making plants highly vulnerable to oxidative damage [24]. It has often been argued that changes in MDA content affect the integrity of the plant cell membrane and that elevated MDA content damages the cell membrane via the process of lipid peroxidation [42]. Therefore, plant cells must consume ROS as signaling molecules and/or regulate their excessive cellular ROS to an optimal balance in order to be non-toxic when ROS levels rise above normal [43].

Photosynthetic pigments are essential components of plants, as they absorb light energy and then transition into a higher energy state [44]. Numerous phytochemical analyses have demonstrated that chlorophyll content is not only a universal indicator of stress conditions but also a crucial biomarker for the photosynthetic capacity of plants [45,46]. From the current study, the observed variation in these pigments (chlorophyll-a, chlorophyll-b and total chlorophyll) at the 1% and 3% PS concentrations reflects that the plant pigmentation system undergoes changes that are characterized as a strategic defense response to various stress signals [47]. In plants, the net photosynthetic rate is a crucial indicator of photosynthetic efficiency, which in turn determines the production of dry matter through improved primary productivity [48]. The observed suppression of chlorophyll content and net photosynthetic rate in response to an increase in PS concentration provides sufficient evidence that they contributed to impaired photosynthetic performance in wheat. The PS stress-induced alterations in plant pigmentation provide additional support for the notion that the generation of excessive ROS could damage chloroplast structures and inhibit photosynthesis [49,50], implying that the wheat antioxidant system is more sensitive to elevated PS concentration. On the other hand, when considering PS shape responses for each photosynthetic parameter individually, we found that PS in the shape of powder has the strongest phytotoxic effects at higher concentrations. This pattern could be due to the unique surface properties of PS powder, which is often described as one of the microplastics with a large specific surface area that can trigger counterproductive changes in chloroplasts and thylakoid structures, ultimately leading to inefficient photosynthetic capacity in stressed plants [51,52].

Measurements of leaf gas exchange offer mechanistic insights into the processes underlying carbon and water fluxes in plant leaves, which in turn enhances the understanding of associated processes across different scales, from single cells to entire ecosystems [53]. It is noteworthy that in our study, increasing the PS concentration constrained leaf carbon and water exchange, leading to synchronous down-regulation of the transpiration rate, stomatal conductance and intercellular CO_2_ concentration. It is to be expected that elevated concentrations of stress factors such as PS disrupt the rate of photosynthesis, because the limitations of photosynthesis can be best recognized by the magnitude of changes in CO_2_ and H_2_O fluxes between the leaf and the atmosphere [54]. Our data also showed that the shape of PS can influence various properties of gas exchange in the leaf, although this is mainly determined by the PS concentration. The observed disruption of gas exchange in the leaf is not surprising if the plants are confronted with toxic suits in the form of microplastic particles in the root zone. The plausible explanation for these results is that PS adhering to the root physically obstructs the root pores to a considerable degree, leading to significant changes in cellular integrity of the root and H_2_O homeostasis, thus restricting water uptake [55]. As a result, this disruption leads to oxidative stress, which then manifests itself in a reduced leaf transpiration rate, impaired stomatal conductance and poor intercellular CO_2_ assimilation [56,57].

## 5. Conclusions

In this study, the effects of PS microplastics on various morphological, physiological and biochemical growth parameters of wheat were investigated in detail. The putative effects of PS microplastics on plant growth parameters were diverse and complex but were mainly determined by the quantitative scale of PS, i.e., its concentration. In addition, the qualitative scale (shape) of PS was only relevant for the detection of phytotoxic effects at higher concentrations. Furthermore, the contrasting effects of PS, either growth-promoting or growth-inhibiting, can also be explained by its structural and physical properties. Nevertheless, targeted redox regulation in plants could be a feasible solution to improve PS stress response in plants. However, improving antioxidant activity might not improve PS tolerance in plants because of their complex and highly time-dependent response to various ROS. Therefore, plant traits such as photosynthetic pigments and leaf gas exchange parameters are of great importance for plant growth to recover under PS stress. Overall, our results show that PS microplastics in different concentrations and shapes could be a potent threat to crops, not only for plant growth and development but also for low crop yields and associated economic losses. They also represent a potentially serious food safety issue that should be investigated by expanding the type, size and concentration of microplastics and extending to large-scale field studies.

## Figures and Tables

**Table 1:** Characteristics of three different shapes of polystyrene microplastics (PS).

Type	Shape	Color	Mean Size (µm)	Density (g/cm^3^)	Zeta Potential (mv)
PS	Powder	White	5–7	0.95	−35
PS	Bead	White	5–10	1.05	−40
PS	Fiber	White	15–25	1.17	−37

**Table 2:** Two-way analysis of variance (ANOVA) of polystyrene plastic shape (PS), plastic rate (PR) and their interaction (PS × R) effects on morphological, root growth, oxidative stress, photosynthesis and leaf gaseous exchange attributes of wheat plants.

Plant Parameters	Plastic Shape (DF = 2)		Plastic Rate (DF = 3)		PS × PR (DF = 6)	
	MS	F-Value	MS	F-Value	MS	F-Value
Plant height (cm)	274.43	25.06 ***	3556.99	324.83 ***	70.37	6.43 ***
Fresh biomass (g pot^−1^)	335.04	41.09 ***	3166.01	388.27 ***	82.70	10.14 ***
Dry biomass (g pot^−1^) LAI (m^2^ m^−2^)	352.78 0.01532	50.68 *** 10.78 ***	3262.19 0.06885	468.64 *** 48.43 ***	91.39 0.00206	13.13 *** 1.45 ^NS^
RL (cm)	35.951	9.82 ***	611.921	167.06 ***	7.763	2.12 ***
RFW (g)	2.6889	12.50 ***	42.0930	195.66 ***	0.4109	1.91 ^NS^
SOD (Unit mg protein^−1^ min^−1^)	23.890	10.30 ***	608.409	262.24 ***	7.816	3.37 ^NS^
POD (Unit mg protein^−1^ min^−1^)	129.70	17.90 ***	1655.64	228.48 ***	39.15	5.40 ***
CAT (Unit mg protein^−1^ min^−1^)	69.728	25.21 ***	829.480	299.92 ***	12.591	4.55 ***
MDA (mmol mg^−1^ protein)	4.3011	84.68 ***	30.5413	601.27 ***	0.6271	12.34 ***
Chl-a (mg g^−1^ FW)	0.30508	38.52 ***	1.22894	155.15 ***	0.17323	21.87 ***
Chl-b (mg g^−1^ FW)	0.04444	10.65 ***	0.56472	135.29 ***	0.08432	20.20 ***
Total Chl (mg g^−1^ FW)	0.56451	27.74 ***	3.43219	168.69 ***	0.49059	24.11 ***
NPR (µmol CO_2_ m^−2^ s^−1^)	16.891	8.07 ***	146.894	70.19 ***	0.750	0.36 ^NS^
TR (mmol H_2_O m^−2^ s^−1^)	0.58377	18.20 ***	4.89884	152.70 ***	0.09277	2.89 ^NS^
SC (µmol H_2_O m^−2^ s^−1^)	0.01335	18.92 ***	0.20735	293.83 ***	0.00229	3.25 ^NS^
Intercellular CO_2_ concentration (µmol mol^−1^)	821.6	80.68 ***	14186.9	1393.21 ***	214.0	21.02 ***

DF = degree of freedom; MS = mean square; F = variance ratio. NS = non-significant; *** = *p* < 0.001. PS = plastic shape; PR = plastic rate; LAI = leaf area index; RL = root length; RFW = root fresh weight; SOD = Superoxide dismutase activity; POD = Peroxidase activity; CAT = Catalase activity; MDA = Malondialdehyde content; Chl-a = Chlorophyll-a; Chl-b = Chlorophyll-b; Total Chl = Total chlorophyll; NPR = net photosynthetic rate; TR = transpiration rate; SC = stomatal conductance.

## Data Availability

The original contributions presented in this study are included in the article. The data presented in this manuscript will be made available by the authors upon request.
